# Optimization of Composite Formulation Using Recycled Polyethylene for Rotational Molding

**DOI:** 10.3390/polym17243290

**Published:** 2025-12-11

**Authors:** Vitaliy Tyukanko, Roman Tarunin, Alexandr Demyanenko, Vladislav Semenyuk, Antonina Dyuryagina, Yerik Merkibayev, Abdigali Bakibaev, Rustam Alpyssov, Dmitriy Alyoshin

**Affiliations:** 1 Department of Chemistry and Chemical Technology, M. Kozybayev North Kazakhstan University, Petropavlovsk 150000, Kazakhstan; vetal3333@mail.ru (V.T.); demianenkoav@mail.ru (A.D.); evdimid@mail.ru (V.S.); adyuryagina@inbox.ru (A.D.); sparkggvpeasy27@gmail.com (R.A.); dvaleshin@ku.edu.kz (D.A.); 2 Department of Metallurgy and Mineral Processing, Kazakh National Research Technical University Named After K.I. Satbayev, Almaty 050013, Kazakhstan; erik_me@mail.ru; 3 Department of Organic Chemistry, Tomsk State University, Tomsk 634050, Russia; bakibaev@mail.ru

**Keywords:** rotomolding, rotational molding, polyethylene recycling, rPE, internal stresses in plastics, ultrasound in plastics, higher harmonics, ESCR

## Abstract

In this work, we optimized three key factors for rotational molding composites: the recycled polyethylene (rPE) content, the pigment (Cp) content, and the process parameter-peak internal air temperature (PIAT). We studied the influence of rPE, Cp, and PIAT on various composite properties. These included mechanical properties (e.g., elastic modulus E), impact strength (MFEsp), surface characteristics (wettability measured by contact angle θ and IR spectroscopy), thermal stability (by DTA–TG analysis), environmental stress cracking resistance (ESCR in hours), and the amplitude of the third harmonic β of the ultrasonic back-wall signal. The IR spectroscopy and contact angle results indicate that adding rPE and pigment slightly increases the composite’s surface hydrophilicity. The results show that PIAT strongly influences all the characteristics of the composites studied. Depending on its percentage, the introduction of rPE can either improve or worsen these composite properties. A correlation was found between β, ESCR, MFEsp, and E, demonstrating that β can serve as a quantitative indicator of internal stress (IS) in rotomolded parts. The recommended optimal composition is rPE 30%, Cp 0.5%, and PIAT 195 °C. Under these conditions, the composite exhibits minimal internal stress and optimal performance, which in turn extends the service life of rotomolded products. Four nomograms were developed: rPE = f(MFEsp, Cp, PIAT) and rPE = f(β, Cp, PIAT), which make it possible to quickly determine MFEsp and β of a product based on the actual PIAT, taking into account rPE and pigment content in the composite (they also allow selecting the rPE and pigment content in the composition depending on the required MFEsp).

## 1. Introduction

At present, the efficient utilization and recycling of plastic waste remain among the most pressing global challenges. Since the invention of Bakelite in the early twentieth century, plastics have become an inseparable part of industrial production and everyday life [1]. Global plastic output has grown continuously since the 1950s, when polyethylene (PE) was first synthesized, and is expected to reach 500–600 million tonnes per year by 2025 [2]. In 2023, worldwide production hit a record 489 million tonnes, yet only 8.17% was recycled [3], highlighting the urgent need for improved recycling infrastructure.

PE is the most widely used thermoplastic, valued for its chemical resistance, low cost, and processability. The global market for virgin PE (vPE) reached about 120 million tonnes in 2022 and is projected to rise to 140 million tonnes by 2025 [4]. However, this growing consumption has led to an intensified accumulation of persistent plastic waste [5,6].

Each year, around 10 million tonnes of plastic enter the environment, contributing to large-scale contamination, including microplastics found even in remote ecosystems [7,8]. The production and incineration of virgin polymers also generate about 4% of global CO_2_ emissions—roughly 2.15 Gt CO_2_-eq annually [9,10,11]. Since the late twentieth century, reports of massive ocean plastic accumulations have revealed that debris not only forms “garbage patches” but also fragments into toxic microparticles affecting marine life and humans [12,13]. Currently, about 60% of plastic waste remains unrecycled and accumulates in landfills or the oceans—around 8 million tonnes annually, a major portion being PE [7]. Yet, owing to its stable molecular structure, PE possesses strong potential for effective secondary recycling [14].

Current strategies to mitigate PE’s environmental impacts remain limited in scale. Roughly 25% of recyclable plastic waste is incinerated with energy recovery—offsetting some energy demand but producing CO_2_ and toxic by-products [15]. Fewer than 1% of plastics undergo chemical recycling (depolymerization or pyrolysis to monomers/hydrocarbon feedstock) due to high capital and energy requirements [16]. About 40% is landfilled, posing long-term risks to soils and waters, while biological degradation accounts for <0.1% because of polymer inertness and the need for prior oxidation [15,16]. Only ~16% is mechanically recycled by shredding, cleaning, and re-melting into secondary pellets [15]. The remaining stream is largely mismanaged: OECD estimates indicate ~22% of global plastic waste enters uncontrolled dumps, open burning, or dispersal outside formal systems [17]. Part of these flows undergoes mechanical–biological treatment (MBT), combining coarse sorting/shredding to extract recyclable fractions with aerobic or anaerobic stabilization of organics to reduce volume and raise the calorific value of residues [18]. Smaller fractions are routed to thermal conversion: pyrolysis yields liquid hydrocarbons (“oil” and waxes) for fuel or feedstock, while gasification converts plastics to syngas (CO + H_2_) for energy or downstream chemical synthesis [19].

Despite the merits of alternative routes, mechanical recycling remains the most economically viable and environmentally safe option for plastics management. It eases pressure on natural resources, reduces landfilling, and lowers greenhouse-gas emissions by substituting primary polymers with secondary ones [20]. Scaled PE recycling could cut demand for virgin PE by about 30% relative to 2019 and further shrink the sector’s footprint [21]. In this context, developing recycled-polyethylene (rPE) formulations for advanced processing is crucial. Rotational molding (RM) stands out as a promising technology for large hollow products that offer uniform wall thickness, low internal stress, and comparatively low equipment costs [22,23]. The method was patented in the UK in the 1930s. It achieved wide industrial uptake after World War II with improved process control and has since gained global traction across automotive, aerospace, chemical, and food industries [24,25]. The RM product market was valued at roughly USD 10 billion in 2020 and is projected to reach about USD 15 billion by 2025 (CAGR ≈ 8–10%), which underscores industrial demand and the method’s potential, particularly for integrating rPE. This momentum has motivated a growing body of research on rPE in RM that is reviewed chronologically below [26].

The first attempt to incorporate recycled polyethylene (rPE) into rotational composites was described in patent WO1993000400A1 (1993), which proposed LLDPE–rHDPE formulations containing up to 90–95% polyethylene, though the exact proportion of the recycled fraction was unspecified [27]. Mixing components with different densities (0.92–0.96 g/cm^3^) produced a synergistic effect, as the Izod impact strength exceeded theoretical values. Further development was presented in US6180203B1 (2001), introducing a two-layer structure with an outer virgin LLDPE barrier and an inner layer of recycled polyolefins [28]. This configuration enabled up to 100% rPE use in inner layers without loss of mechanical or chemical performance. A major step forward came with WO2016102341A1 (2016), which incorporated crosslinked polyethylene (XLPE) waste in amounts of 10–30% [29]. The resulting materials maintained molding stability, with flexural modulus above 840 MPa (up to 1000–1200 MPa) and tensile strength between 15 and 20 MPa at elongation up to 5%. Since 2020, patent activity in rPE-based rotational molding has intensified markedly—rising from 3 patents (1993–2016) to 7 between 2020 and 2025. Notably, the Chinese patent CN109664584B (2020) proposed a one-step process for producing foamed rPE products with impact strength of 74–85 J, significantly exceeding that of virgin PE (14–33 J) [30]. WO2021222984A1 (2021) proposed multilayer constructions in which rPE layers 3–10 mm thick were combined with vPE, achieving a balance between processability—MFI 1–10 g/10 min and density 930–970 kg/m^3^—and impact strength [31]. CA3190761A1 (2021) reported rotational molding compositions containing 10–50 wt.% rPE, with a 20% example and an overall vPE/rPE ratio up to 80/20, yielding an internal rPE layer with controlled roughness of about 6.3 µm that improved durability and coating adhesion [32]. Recent developments in US20230093454A1 and US20230124453A1 (2023) focus on localizing rPE at the inner surface, enabling roughness of 4–30 µm and promoting carbonyl group formation that enhances adhesion to polyurethane coatings [33,34]. US20230339150A1 (2023) introduced spontaneous phase segregation that forms an internal surface with 12–20 µm roughness and high adhesion to polar materials [35]. Concluding this sequence, WO2025114813A1 (2025) disclosed a three-component composition of 60–80% MDPE, 5–25% HDPE, and 10–30% rPE that delivers impact strength of at least 25 J/m and in some cases above 50 J/m, confirming the feasibility of substantial rPE incorporation without sacrificing performance [36].

Unlike patent sources, which emphasize technological feasibility and industrial applicability, articles indexed in Scopus and WoS systematically analyze the ranges of rPE content and their correlation with mechanical properties (strength, Young’s modulus, and impact toughness), thereby providing an empirical foundation for further optimization of RM compositions [25]. Pick et al. [23] demonstrated that manufacturing containers from 100% rPE (recycled tanks) was accompanied by an increase in viscosity at low shear rates, a decrease in impact strength (0.5–1 J/mm versus 7.2–17 J/mm for vPE), and powder-quality issues. In the work of Cestari et al. [37], 50/50 vPE/rHDPE blends from bottles, pipes, and household waste provided impact strength up to 9.3 J/mm under compression molding, but in RM conditions, impact strength dropped by 85–87% and flexural modulus decreased by 20–30%. Díaz et al. [38] investigated the inclusion of cable waste up to 50 wt%, finding that concentrations above 35% led to significant degradation of strength and Young’s modulus, while elongation at break dropped sharply even at 10% recycled content. Chaisrichawla and Dangtungee [39] noted that adding rHDPE (0–50 wt%) to LLDPE reduced tensile strength but increased impact toughness and Young’s modulus, showing a positive effect at 10% rPE. Other studies confirmed the possibility of using 100% rHDPE or PP/rHDPE blends in building blocks, although lower mechanical performance was observed and the introduction of additives was required (e.g., 5 wt% Al_2_O_3_ to achieve UL-94 V0) [40]. Research on rLDPE [41] and PLA [42] also showed significant reductions in strength and impact resistance when 100% recycled plastic was used in the composition. An additional direction involved adding 20–50 wt% of ground rubber into LDPE, which resulted in elastomer-like composites with elongation at break >100%, but at higher concentrations the properties deteriorated [43]. Finally, Dou and Rodrigue [44] demonstrated the feasibility of producing foamed products based on 100% rHDPE using chemical foaming agents, although this reduced thermal conductivity and increased cell size.

The surveyed patents and Scopus/WoS studies collectively indicate that rotational molding is a technologically versatile process and that rPE can be effectively integrated into RM formulations [45,46,47]. Yet, most reported recipes target narrowly defined industrial needs, and the field still lacks a unifying theoretical framework that links rPE processing conditions to the performance of molded parts. Uncertainty about the optimal rPE content continues to drive variability in rheology, uneven additive distribution, and elevated risks of micro-voids, internal stresses, and delamination [45,46,47]. These risks become critical for containers that hold aggressive liquids, where chemical exposure accelerates degradation [48]. Foundational work by F. P. Gomes attributes residual stresses in RM to temperature and pressure gradients during melt cooling and crystallization, and ultrasonic experiments confirm a correlation between the third-harmonic amplitude of the bottom signal and the levels of plastic deformation and residual stress [49,50]. Follow-up studies by Gomes and co-authors show that heat treatment and swelling can reshape stress-relaxation behavior, which opens pathways for targeted control [50]. Industry still relies on standardized diagnostics such as crack-resistance testing in aggressive media under ASTM D1693 and FNCT under ISO 16770, although test durations approaching 1500 h constrain rapid quality assurance [51,52]. Against this backdrop, ultrasonic testing is emerging as a nondestructive, real-time tool, with third-harmonic analysis enabling accurate localization of defective zones in rotationally molded polyethylene and case studies on tanks demonstrating feasibility for in-process monitoring and life-extension strategies [52].

This research—developing optimal rotational molding formulations that integrate recycled polyethylene (rPE) and ultrasonic testing (UT)—is highly relevant due to technological, environmental, and economic factors. The use of rPE significantly reduces material costs, while UT allows for rapid evaluation of residual stresses, ensuring product durability and reliability. This combination strengthens the competitiveness of enterprises that use rotational molding for plastic recycling. In this study, an industrial composite was developed by varying the rPE content, pigment concentration, and processing parameters defined by the peak internal air temperature (PIAT) indicator. For the first time, we applied ultrasonic testing to optimize an industrial RM composite’s composition and to study the influence of rPE on residual stresses in parallel with the conventional environmental stress crack resistance (ESCR) metric. The research focused on containers designed for aqueous surfactant solutions, where stress cracking is a key degradation mechanism. Understanding how rPE content affects residual stresses also provides insights into microstructural evolution in semicrystalline polymers. The proposed approach combines formulation optimization with modern nondestructive testing, improving both mechanical strength and impact toughness while reducing internal stresses. This outcome gives the study clear environmental and economic significance and aligns it with the goals of sustainable industrial development and the circular economy [53,54]. According to the European Environment Agency, the use of recycled polyethylene makes it possible to reduce the demand for primary raw materials by 20–60% and to decrease material losses over the product life cycle [53]. The relationship under investigation between the content of recycled polyethylene, the amount of black pigment, residual stresses, and ESCR characteristics is of fundamental importance for assessing the service reliability of rotomolded products. Optimization of the composition and reduction in residual stresses extend their actual service life and reduce raw material consumption [54]. These effects are consistent with UN Sustainable Development Goal 12 “Responsible Consumption and Production”, including Targets 12.2 and 12.5 aimed at improving resource efficiency and reducing waste.

The combined effects of rPE content, pigment concentration, and RM parameters (PIAT) on residual stresses and mechanical performance are complex, so a probabilistic–deterministic design method was employed. This method reduces the number of required experiments and captures the interdependence of key factors through multiparametric mathematical models, enabling targeted optimization of the composite according to residual-stress thresholds and impact strength requirements [55,56,57,58,59,60,61,62,63,64,65].

## 2. Materials and Methods

### 2.1. Materials

For the preparation of the samples, hexene-based linear medium-density vPE (LLPE, Lupolen 4021 K RM) with a density of 939.5 kg/m^3^ and a melt flow index (MFI) of 4 g/10 min (190 °C/2.16 kg) from LyondellBasell (Wesseling, Germany) was used. After its primary processing (container caps), rPE Lupolen 4021 K RM was also obtained. As a pigment, ungranulated (powder form) low-activity technical carbon black, grade P803 according to State Standard GOST 7885-86, supplied by Tuymazytekhuglerod (Tuymazy, Russia), was employed. As a mold release agent, the industrial product MODENGY^®^1014 (Bryansk, Russia) was used.

### 2.2. Samples Preparation

The samples were manufactured using a shuttle-type rotational molding machine, model FD4.0, produced by Yantai Fangda Rotational Molding Co., Ltd. (Yantai, China), equipped with three cubic molds (dimensions 500 mm × 500 mm × 500 mm). After loading 10 kg of polyethylene and the required amount of pigment, the closed mold was placed into the heating chamber of the machine, which had been preheated to 340 °C. To ensure uniform distribution of the pigment within the polymer matrix, the mixture was blended for 5–10 min using a VCG-150 mixer manufactured by Yantai Fangda Rotational Molding Co., Ltd. (Yantai, China). The mold was kept in rotation for 25–35 min at 300–340 °C (heating cycle). The internal air temperature (IAT) was monitored with a thermocouple through the mold’s vent during the entire process. The molds were heated to the required PIAT, after which heating was stopped and the cooling process was initiated. Once the internal air temperature IAT decreased to 90 °C, the samples were removed from the mold. During the rotational molding process (heating and cooling), the rotation speed for the major axis (arm speed) and the minor axis (plate speed) was set at 5 rpm and 9 rpm, respectively.

### 2.3. Ultrasonic Testing Method

**Figure 1:**
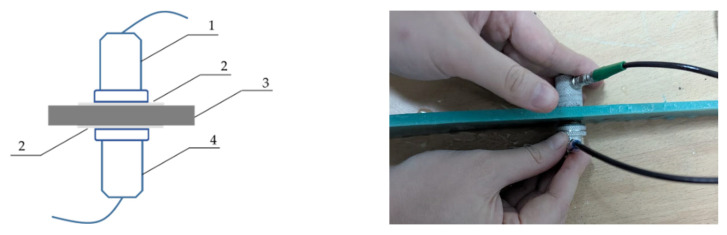
Shadow method of ultrasonic flaw detection (1—transmitting piezoelectric transducer; 2—glycerin layer; 3—experimental plastic sample; 4—receiving piezoelectric transducer).

A previous study [52] measured the height of the third harmonic (β) in PE samples using a mirror-shadow ultrasonic testing method. In our research, the simplified shadow method proved to offer the most stable measurements. In the shadow method (used here with a UCD-60N ultrasonic flaw detector), two piezoelectric transducers are placed on opposite sides of the glycerin-coated plastic sample, symmetrically positioned relative to it (Figure 1).

An electrical pulse with an amplitude of 200 V, a carrier frequency of 2.5 MHz, and a repetition frequency of 20 Hz was applied to the upper piezoelectric emitter of the flaw detector. After a single passage through the plastic, the elastic ultrasonic oscillations were recorded as variations in the back-wall echo intensity at the opposite (receiving) transducer. To determine the coefficient β, the “Spectrum” function of the flaw detector was employed.

**Figure 2:**
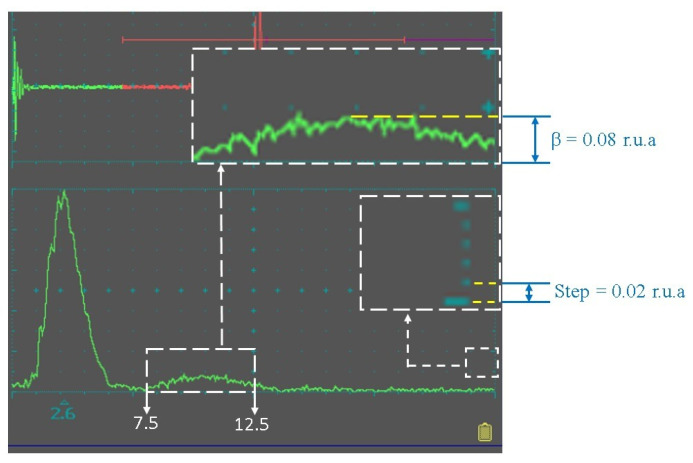
Determination of the third harmonic amplitude of the bottom signal using the UCD-60N ultrasonic flaw detector.

On the spectral coordinate grid of the flaw detector, the amplitude axis (Oy) comprises 10 cells, each divided into 5 subdivisions with a step size of 0.02 relative units (r.u.a.). The amplitude of the first harmonic always occupies the full range of these 10 cells, corresponding to 50 subdivisions of the spectrometric scale. The value of the coefficient β is determined on the instrument scale, where each subdivision represents 0.02 r.u.a. By finding the peak subdivision count on the Oy-axis in the 7.5–12 MHz range, β can be determined with a maximum absolute error of ±0.01 (in relative arbitrary units, r.u.a.) (see Figure 2).

### 2.4. Mechanical Testing Method

**Figure 3:**
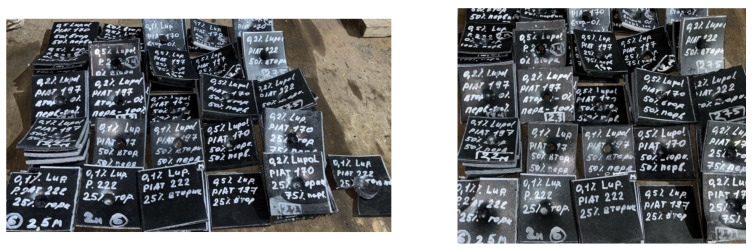
Samples of the studied composites after determination of specific Mean Failure Energy (MFE_sp._).

From the fabricated plastic cubes (500 mm × 500 mm × 500 mm), two types of specimens were cut for mechanical testing (Figure 4 in [52]). The first view of the samples (upper right) was used to determine the tensile properties of the material, i.e., the flexural modulus of elasticity (E, MPa), the yield tensile stress (σ_yield_, MPa), and the ultimate tensile stress at break (σ_break_, MPa), in accordance with ISO 527-2:2012, at a speed of 5 mm/min. Tensile tests were carried out on a testing machine from Haida international equipment Co., Ltd., HD-B617-S (Dongguan, China). Samples for mechanical testing were tested at temperature (20 ± 2)°C. Their holding time in the oven (to equalize the temperature in the plastic) was 24 h. The modulus and strength values given are based on the average of at least 18 samples. A second type of specimen (125 mm × 125 mm plate) was used to characterize the impact material, and Mean Failure Energy (MFE) was determined according to the ARM Low Temperature Impact Test standard [66] (see Figure 3).

The MFE tests were carried out on a test rig constructed according to the drawings on page 9–12 of the “Low Temperature Impact Test. Association of Rotational Molders International (ARM)” [66] at 20 ± 1 °C. Samples for the determination of MFE were kept at a temperature of minus (40 ± 1) °C for 24 h in a freezer. The reported values of MFEsp. are based on the average of at least eighteen specimens and are given in J/mm, found using the following Formula (4):

(1) $$MFE_{sp}=\;\frac{MFE}{h}$$

where *h* is the sample thickness, in mm; *MFE_sp_*_._—specific Mean Failure Energy, J/mm; *MFE*—Mean Failure Energy, J.

Sample density was measured based on the Archimedes method as described in International Organization for Standardization (ISO) 1183 on an Electronic Densimeter, model MD-300S (Osaka, Japan).

### 2.5. ESCR Test Method

**Figure 4:**
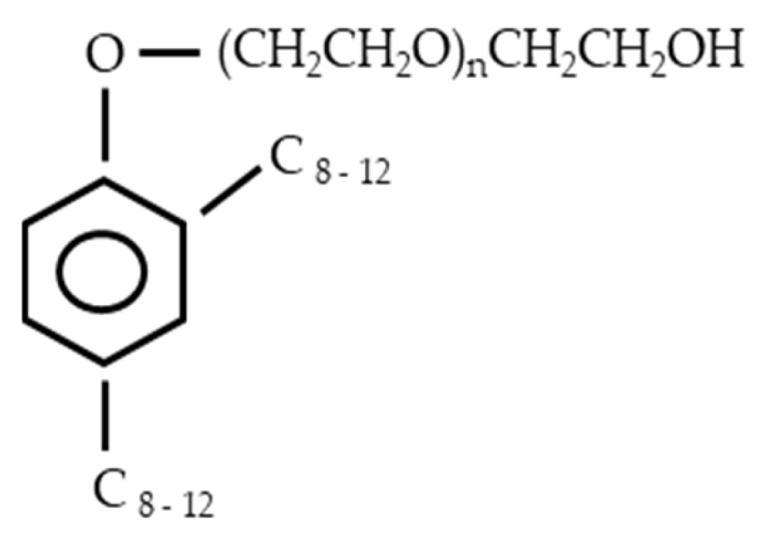
Structural formula of the nonionic surfactant OP-7.

The environmental stress crack resistance (ESCR) time was determined in accordance with ASTM D1693 (Bent-Strip, the so-called Bell test), Condition “C” at (100 ± 0.5) °C (accelerated testing), using a ZL-3046 instrument (Dongguan, China). The testing medium was a 10% aqueous solution of OP-7 (a nonionic surfactant, functional analog of Igepal CO-630). OP-7 represents the products of ethylene oxide treatment of a mixture of mono- and dialkylphenols; its structural formula is shown in Figure 4.

OP-7 was chosen as the testing surfactant (instead of the reagent Igepal CO-630 recommended by ASTM D1693) because it is widely used as a wetting agent in major industrial pesticides within the Republic of Kazakhstan. These pesticides must be stored and transported in tanks manufactured from the studied composites. Therefore, ESCR was evaluated using an industrially applied surfactant.

**Figure 5:**
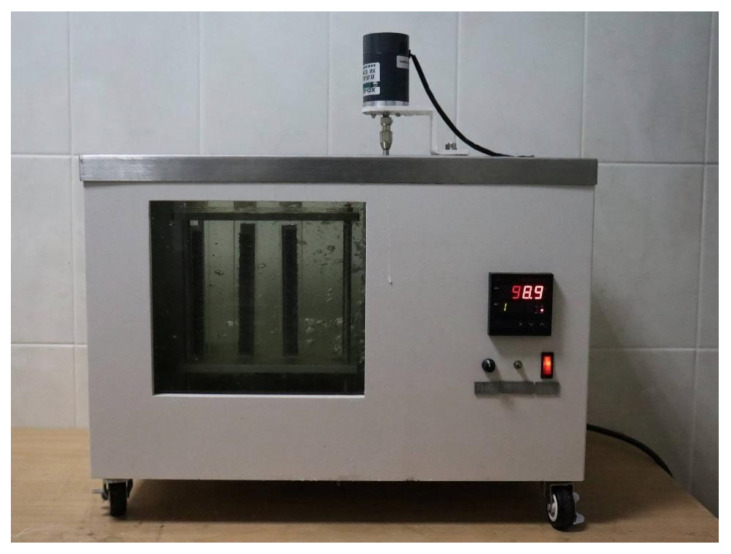
Photographs of several composite formulations during ESCR testing.

The criterion for the onset of ESCR in the composites was the complete failure of all test plates (100%)-F100 (see Figure 5).

### 2.6. Contact Angle Measurement Method

The contact angle (θ°) of the composites was determined using an ACAM instrument (Kolkata, India) with the standard software package Apex Acam Software, version 2.026.088.1. The PE sample was fixed on the stage and leveled relative to the baseline within the camera’s field of view. Using a dispenser, a drop of distilled water was carefully formed above the specimen surface and gently placed onto it, avoiding any inertial spreading. After the drop stabilized, its side profile was recorded.

The calculation of θ was performed using the elliptical method: reference points were manually placed along the contour of the drop profile in the program interface (arbitrary number, but no fewer than six), after which the software approximated the profile with an ellipse and automatically calculated the contact angles as the tangents to the approximating curve at the three-phase line. For each position, left and right angles were recorded, and the software reported the mean value for the given drop. Measurements were carried out at three different locations on each sample, and at each location, three independent drops were deposited and analyzed. The article presents average values from three measurements at three locations (a total of nine determinations per specimen).

The approach employed was the sessile drop method with elliptical profile approximation and tangent-based calculation of θ at the point of triple contact.

### 2.7. Fourier Transform Infrared Spectroscopy Method

Fourier-transform infrared (FTIR) spectra were obtained using an InfraLUM FT-08 spectrometer equipped with an ATR accessory (Madison, WI, USA), operating in the 400–4000 cm^−1^ range with 25 scans and a resolution of 4 cm^−1^.

### 2.8. Method of Thermal Analysis

Thermal analysis was performed using a NETZSCH STA 409 PC/PG differential thermogravimetric analyzer, which enables simultaneous recording of thermogravimetric (TG), differential thermal (DTA), and derivative thermogravimetric (DTG) curves. Data acquisition and processing were carried out with the NETZSCH Proteus software package, version 5.2.1. Measurements were carried out in the standard DTA-TG mode. A platinum–rhodium (Pt-Rh) crucible, characterized by high chemical and thermal resistance, was used. The experiment was conducted in an inert gas atmosphere—nitrogen (N_2_)—supplied at a constant flow rate of 50 mL/min. The heating temperature range was 20 °C to 600 °C, with a uniform heating rate of 30 °C/min. The obtained data include the following curves: TG—reflecting the mass change as a function of temperature; DTG—the derivative curve showing the rate of mass change; DTA—registering thermal effects (endothermic and exothermic processes) occurring in the studied sample. For each sample, the following parameters were determined: the onset temperature of thermal degradation (Tb, °C), the temperature of maximum mass loss (Tmax, °C), and the percentage of sample mass loss (M, %).

### 2.9. Experimental Design

#### Planning an Experiment for Modeling

Modeling of the combined influence of the rPE content, pigment concentration (Cp), and the technological parameter PIAT on the indicators of mechanical and thermal properties of the composites, the third harmonic amplitude (β, r.u.a.) of the ultrasonic signal, impact strength (MFEsp., J/mm), contact angle, and ESCR was carried out using the probabilistic–deterministic planning (PDP) method. The complete algorithm and a detailed description of the PDP method for modeling multiparametric processes are presented in studies [55,56,57,58,59,60,61,62,63,64,65].

An orthogonal experimental plan for three input parameters, each varied at three levels, was developed on the basis of a Latin square in accordance with the PDP methodology. In the modeling using the PDP method, the following steps were performed:

1. Input factors and their levels were defined as follows:
  - rPE content: 0%, 25%, 50%;
  - Pigment (Cp) content: 0.1%, 0.2%, 0.5%;
  - PIAT: 170 °C, 197 °C, 222 °C.
2. Next, an orthogonal plan of a three-factor experiment at three levels was developed. Table 1 presents this orthogonal plan and the results of the experimental determination of the mechanical and thermal properties of the composites, the ultrasonic signal amplitude (β), MFEsp., contact angle, and ESCR.

## 3. Results

### 3.1. The Influence of Optimization Parameters (rPE, Cp, and PIAT) on the Amplitude of the Ultrasonic Third Harmonic β (r.u.a.)

**Figure 6:**
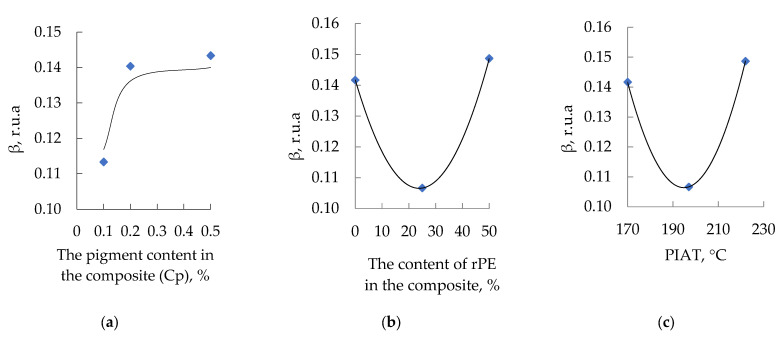
Influence of rPE content (**a**), pigment content (**b**) and PIAT (**c**) on β at 20 ± 1 °C.

The influence of rPE content, pigment (Cp), and the technological parameter PIAT is shown in Figure 6.

The influence of the studied optimization parameters on the amplitude of the third harmonic of the ultrasonic signal is not straightforward. The dependences of β on the rPE content and PIAT are generally identical. An increase in the pigment content in the composite up to 0.2% leads to an increase in β until saturation is reached at 0.140–0.145 r.u.a.

### 3.2. The Influence of Optimization Parameters (rPE, Cp, and PIAT) on the Mechanical Properties of the Composites

**Figure 7:**
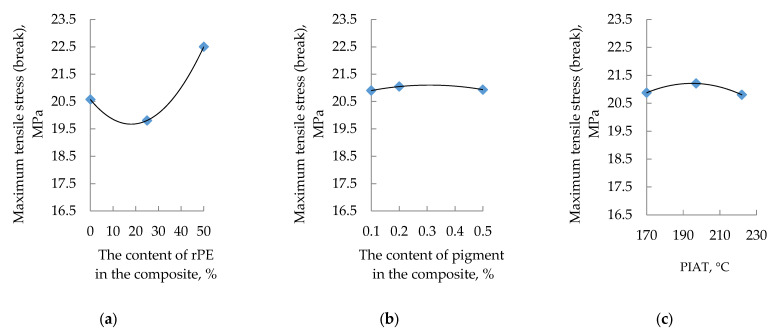
Influence of rPE content (**a**), pigment content (**b**) and PIAT (**c**) on the maximum tensile stress (break) at 20 ± 1 °C.

The influence of rPE content, pigment content, and PIAT on the maximum tensile stress at break is shown in Figure 7.

**Figure 8:**
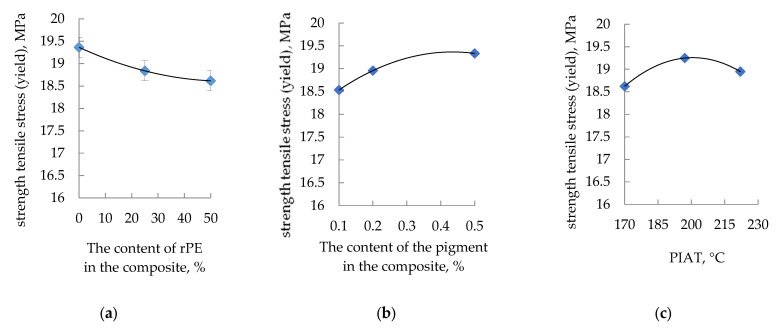
Influence of rPE content (**a**), pigment content (**b**) and PIAT (**c**) on the maximum tensile stress (yield) at 20 ± 1 °C.

The influence of rPE content, pigment content, and PIAT on the tensile yield stress at yield is shown in Figure 8.

**Figure 9:**
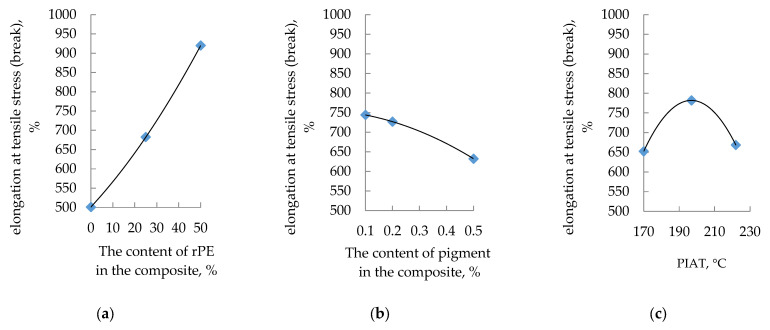
Influence of rPE content (**a**), pigment content (**b**) and PIAT (**c**) on the elongation tensile stress (yield) at 20 ± 1 °C.

The influence of rPE content, pigment content, and PIAT on the elongation at tensile stress at break is shown in Figure 9.

**Figure 10:**
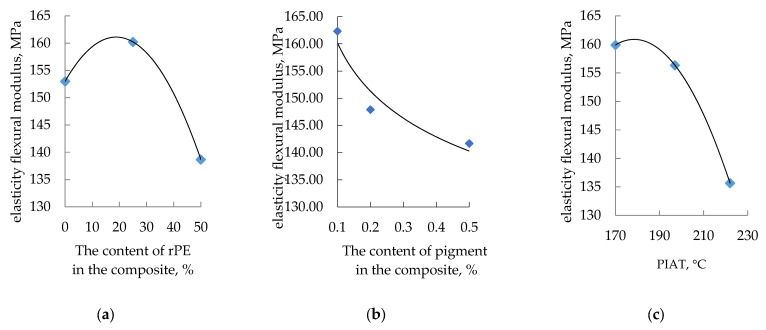
Influence of rPE content (**a**), pigment content (**b**) and PIAT (**c**) on the elasticity flexural modulus at a temperature of 20 ± 1 °C.

The influence of rPE content, pigment content, and PIAT on the elastic modulus is shown in Figure 10.

The influence of the studied optimization parameters on the mechanical properties of the rotationally molded composites is not straightforward. In general, as the rPE content in the composite increases, the yield strength decreases, the tensile strength stabilizes and then increases (at rPE contents above 30%), the strain at tensile strength increases, and the elastic modulus exhibits a maximum (at rPE contents above 30%). With increasing pigment content, yield strength and tensile strength both rise, whereas elongation at break decreases. The elastic modulus initially drops (up to a pigment content of ~0.2%) and then levels off. In contrast to the previously considered optimization parameters, unlike rPE and pigment content, the processing parameter PIAT has the same effect on all the mechanical properties. At PIAT ≈ 197 °C, the composites exhibit peak mechanical performance, whereas at the lower (170 °C) and higher (222 °C) PIAT extremes, all properties decline (except the elastic modulus; see Figure 10c).

### 3.3. The Influence of Optimization Parameters (rPE, Cp, and PIAT) on the Impact Strength of the Composites

**Figure 11:**
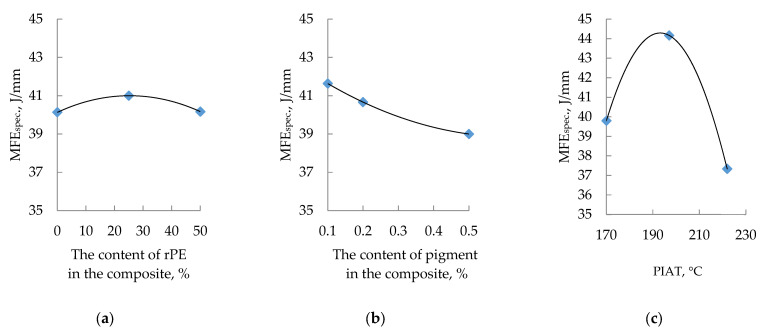
Influence of rPE content (**a**), pigment content (**b**) and PIAT (**c**) on the specific Mean Failure Energy (MFE_sp._) at a temperature of 20 ± 1 °C.

The influence of rPE content, pigment (Cp), and the technological parameter PIAT on the specific mean failure energy (MFEsp., J/mm) is shown in Figure 11.

As the rPE content and the processing parameter PIAT increase, the impact strength of the composites rises to a maximum (at rPE = 30% and PIAT = 197 °C), and then MFEsp begins to drop off.

### 3.4. The Influence of Optimization Parameters (rPE, Cp, and PIAT) on the Wettability of the Composites (Contact Angle θ)

**Figure 12:**
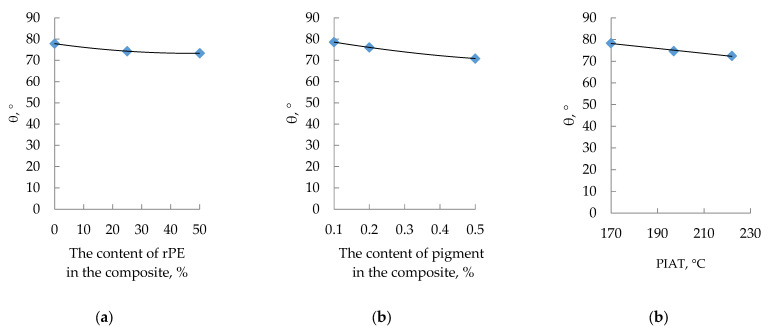
Influence of rPE content (**a**), pigment content (**b**) and PIAT (**c**) on the contact angle of wettability (θ°).

**Figure 13:**
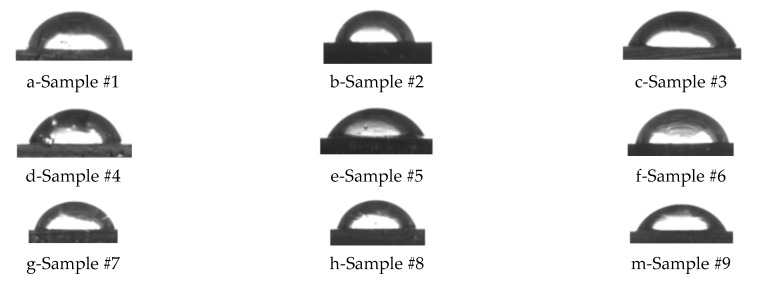
Photographs of distilled water droplets on the nine investigated composite formulations.

The influence of rPE content, pigment content, and the technological parameter PIAT on the contact angle of wettability (θ) is shown in Figure 12 and Figure 13.

As the contents of rPE and pigment and the processing parameter PIAT increase, the contact angle decreases, indicating a reduction in the composite’s hydrophobicity (higher surface wettability).

### 3.5. The Influence of Optimization Parameters (rPE, Cp, and PIAT) on the ESCR of the Composites

**Figure 14:**
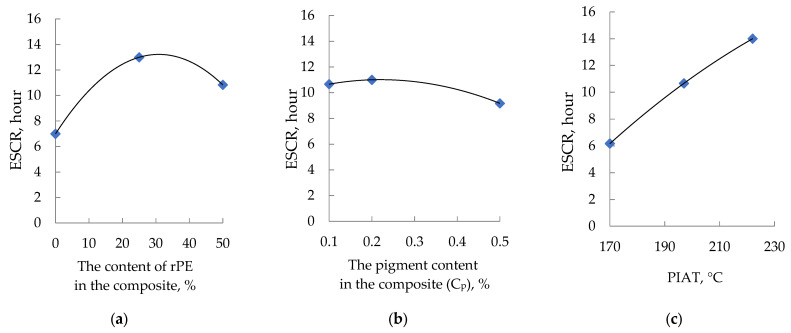
Influence of rPE content (**a**), pigment content (**b**), and PIAT (**c**) on the ESCR of the composites.

The influence of rPE content, pigment (Cp), and the technological parameter PIAT on the ESCR (h) of the composites is shown in Figure 14.

Increasing rPE and pigment contents raises the ESCR to an optimum value, after which it declines. By contrast, increasing PIAT causes a near-linear increase in ESCR.

### 3.6. The Influence of Optimization Parameters (rPE, Cp, and PIAT) on the Density of the Samples

**Figure 15:**
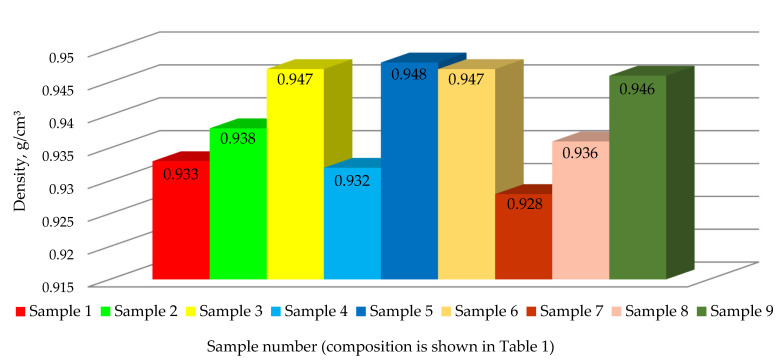
Experimentally determined density of nine polyethylene samples (at a temperature of 20 ± 1 °C).

The experimentally determined density of the samples is shown in Figure 15.

Samples molded at the low PIAT of 170 °C showed the lowest density (0.928–0.933 g/cm^3^). Samples molded at a normal PIAT (≈197 °C) or high PIAT (222 °C) attained higher densities (0.938–0.948 g/cm^3^) and are considered acceptable for industrial use.

### 3.7. IR Spectroscopy of the Obtained Samples

**Figure 16:**
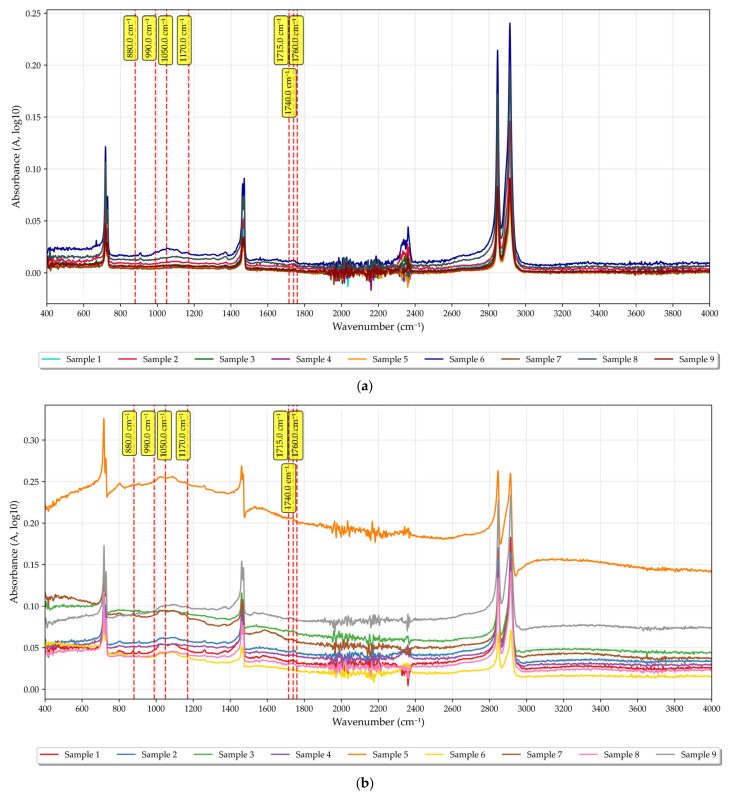
IR spectra of the nine investigated formulations of rotational polyethylene ((**a**)—lateral surfaces of the samples; (**b**)—cross-section of the sample wall).

The obtained IR spectra of the samples are illustrated in Figure 16.

All nine composite samples exhibited very similar IR spectra, regardless of rPE content, pigment content, or PIAT. Notably, increasing rPE, pigment content, or PIAT did not lead to any visible increase in the intensity of bands associated with oxygen-containing groups (markers of thermo-oxidative degradation):

1710–1720 cm^−1^: keto carbonyls (C=O) in oxidized PE, 1735–1745 cm^−1^: ester carbonyls (C=O)–oxidation products (ether/lactone fragments) [67];

1760–1765 cm^−1^: high-frequency shoulder of the general carbonyl region of PE oxidation products;

1170–1050 cm^−1^: C–O/C–O–C vibrations in oxidized fragments [68];

990–880 cm^−1^: out-of-plane =CH deformations (vinylene/vinyledene bonds)—indicators of unsaturation [67].

### 3.8. The Influence of Optimization Parameters (rPE, Cp, and PIAT) on the Thermal Properties of the Composites

The obtained DTA and DTG thermograms of the nine investigated samples are presented in Supplement Figure S1.

**Figure 17:**
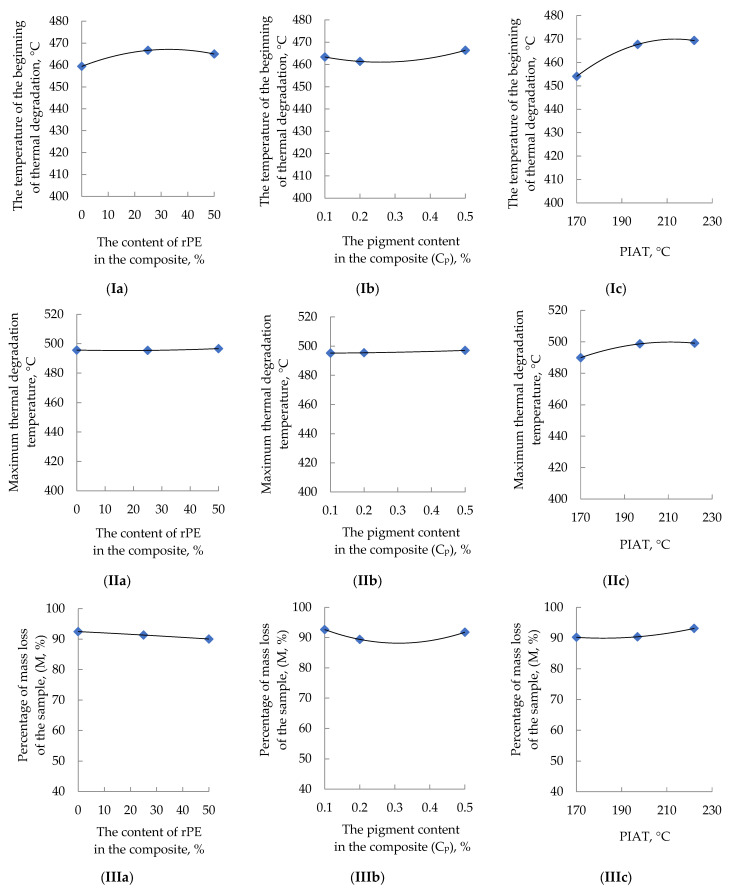
Influence rPE content (**a**), pigment content (**b**), and PIAT (**c**) on the onset (beginning) degradation temperature (°C) (**I**), the temperature of maximum mass loss (°C) (**II**), and the percentage of mass loss of the sample (M, %) (**III**).

The influence of rPE content, pigment content, and PIAT on the thermal properties of the composites is shown in Figure 17.

The effects of the optimization parameters on composite thermal properties are nonlinear. Increasing rPE content raises both the onset (T_b_) and peak (T_max_) degradation temperatures, while reducing the total mass loss M. Increasing pigment content raises T_b_ and causes M to decrease initially and then increase at higher pigment loadings. A higher PIAT generally improves the composites’ thermal stability.

### 3.9. Development of a Mathematical Model

In connection with the well-known effect in rotomolding practice of reduced impact strength in products manufactured from 100% rPE, and at the request of the technical department of the partner organization of this study, AVAGRO Ltd. (Petropavlovsk, Kazakhstan), mathematical modeling was carried out for the key parameters, determining the service life of rotationally molded products—MFE and β. Considering the reduced impact strength observed in products made from 100% rPE (a concern raised by our industry partner, «AVAGRO» Ltd. (Petropavlovsk, Kazakhstan)), we carried out mathematical modeling for the key service-life parameters MFE and β. The experimental data for each combination of input factors (Table 1) were plotted as response surfaces following standard PDP procedures [55,56,57,58,59,60,61,62,63,64,65]. This yielded the partial dependence curves shown in Figure 6, Figure 7, Figure 8, Figure 9, Figure 10, Figure 11, Figure 12, Figure 14 and Figure 17.

To derive a multifactorial statistical mathematical model of the effect of rPE, pigment content (C_p._) and PIAT, the equation proposed by M. M. Protodiakonov (2) was used:

(2) $$Y_{0}=\frac{\prod_{i=1}^{n}Y_{i}}{Y_{M}^{n-1}}$$

where $Y_{0}$ is the generalized equation; $Y_{i}$ is the particular function; $\prod_{i=1}^{n}Y_{i}$ is the product of all particular functions; *n* is the number of particular functions equal to the number of input parameters; and $Y_{M}^{n-1}$ is the total average of all the considered values of the generalized function to a degree one less than the number of particular functions.

Using the obtained partial dependencies, shown in Figure 6 and Figure 11, multifactor mathematical models (3, 4) were built based on the generalized Protodyakonov Equation (2):

(3) $$MFE_{sp}=\frac{\left(a\cdot rPE^{2}+b\cdot rPE+c\right)\cdot \left(d\cdot C_{P}^{2}+e\cdot C_{P}+f\right)\cdot \left(g\cdot PIAT^{2}+h\cdot PIAT+i\right)}{Y_{M}^{2}},\;J/\mathrm{mm}$$

(4) $$\beta =\frac{\left(a\cdot rPE^{2}+b\cdot rPE+c\right)\cdot \left(d\cdot \left(1-e^{e\cdot C_{P}}\right)\right)\cdot \left(g\cdot PIAT^{2}+h\cdot PIAT+i\right)}{Y_{M}^{2}},\;r.u.a.$$

Table 2 presents the values of the coefficients included in Equations (3) and (4).

The reliability of the obtained mathematical models was estimated by calculating the coefficients of nonlinear multiple correlation. The goodness of fit of the models is very high: even the lowest nonlinear multiple correlation coefficient achieved was 0.86.

## 4. Discussion

**Figure 18:**
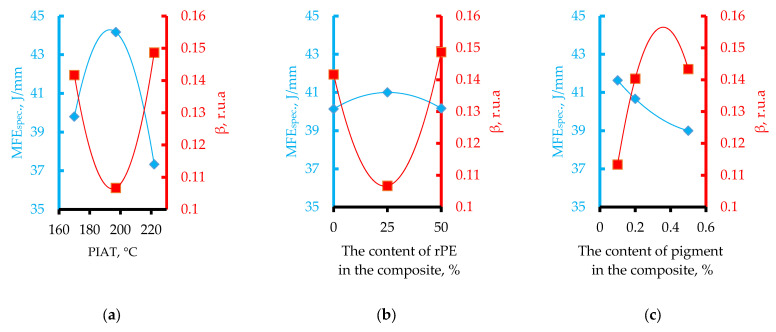
Correlation between MFEsp. and the β for rotational composites as a function of the technological parameter PIAT (**a**), rPE content (**b**), and pigment content (Cp) (**c**).

The effect of PIAT on β correlates with the well-known practical relationship between PIAT and part impact strength (MFE_sp_), as shown in Figure 18a.

The curve dependence β = ƒ(PIAT) is essentially the inverse of the classical MFEsp = ƒ(PIAT) dependence well known in rotational molding (Figure 18a) [19]. As PIAT increases from 170 °C to about 195–205 °C, β decreases by ~20% and then levels off, while the impact strength reaches an optimum (MFEsp stays above 42 J/mm in this range). At PIAT ≈ 220 °C, β rises again (by ~36%) and MFEsp drops by ~15%.

One possible explanation for the observed correlation between β, PIAT, and MFE is that the internal stress (IS) in rotomolded parts is quantitatively linked to β. This assumption is supported by F.P.C. Gomes and M.R. Thompson [50], who showed that the normalized third-harmonic amplitude β in rotomolded products strongly correlates with their internal stresses. Previously published studies [52] examined ultrasonic NDT methods for assessing the quality of rotomolded products made from Lupolen 4021 K RM and other hexene-based linear PEs. Notably, those studies used a different pigment (Heubach Vynamon Green 600734 (PG7)) instead of the low-activity carbon black grade P803 (Tuymazytekhuglerod, Tuymazy, Russia) used here. The samples with green pigment exhibited significantly less ultrasonic attenuation than those with black pigment. Thus, in earlier tests with green-pigmented samples, we successfully employed echo-pulse (single transducer) and mirror-shadow (dual transducer) methods with one-sided access. However, for the black-pigmented samples, these methods proved impractical due to the high attenuation, and therefore the through-transmission shadow method (with transducers on both sides of the sample) was adopted. When testing green samples by the echo pulse method, it was found that in “low PIAT” samples, the attenuation of the ultrasonic signal is significantly stronger compared to “normal PIAT” and “overheated PIAT”. In the last two types of samples, the attenuation was approximately the same [52]. When testing black plastics using the shadow method, there was no statistically significant difference in the attenuation of the ultrasonic signal depending on the PIAT value. The effect of PIAT on the spectral characteristics of the ultrasonic signal transmitted through the test sample in green and black plastics has one common feature and several significant differences observed: the value of the third harmonic increases with increasing PIAT from “normal” to “overheated”—this is the similarity between green and black plastic; there is strong noise in the ultrasonic spectrum of the ‘low PIAT’ green plastic, preventing determination of the third harmonic amplitude (assumed to be zero in this case) [52]; in contrast, the “low PIAT” black plastic still exhibited a measurable third harmonic, with an amplitude nearly as high as that of the “overheated PIAT” condition. In preliminary tests, spectra of black-pigmented plastic showed no appreciable harmonic noise from 20 °C up to 80 °C. By contrast, the green plastic exhibited increasing noise with temperature, and by 80 °C, the noise was so strong that the third harmonic amplitude could not be determined at any PIAT [52]. Therefore, for green plastic the third harmonic measured at 20 °C was used as the quality criterion for comparing PIAT conditions [52].

**Figure 19:**
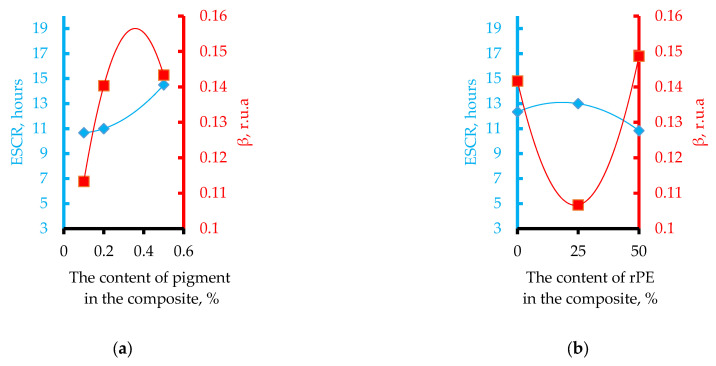
Correlation between ESCR and β for rotational composites as a function of rPE content (**a**) and pigment content (**b**).

To verify this assumption, we conducted an additional study of internal stresses in the obtained samples. A widely used measure of internal stress in polyethylene is the Environmental Stress Crack Resistance (ESCR) per ASTM D1693. This parameter is routinely listed in data sheets for any industrial PE grade intended for liquid storage applications (pipes, tanks, fittings, etc.). In our experiments, we found that ESCR correlates with β as functions of rPE and pigment content (Figure 19).

As rPE content increases to ~25–30%, β decreases while ESCR rises—confirming that internal stresses in the composite are reduced. Thus, β can serve as a quantitative measure of internal stress in rotomolded parts. For example, increasing PIAT from 170 °C to ~195–205 °C lowers internal stress (β) by promoting a more equilibrated polymer structure, which in turn yields higher impact strength. However, further raising PIAT above ~225 °C initiates thermo-oxidative degradation (as demonstrated in the works of F.P.C. Gomes [50]). At this stage, carboxyl groups appear on the surface of PE (identified by the peak at 1740 cm^−1^), leading to a sharp rise in internal stress and a corresponding drop in impact strength [53]. Similarly, raising the rPE content up to 30% lowers internal stress by about one-third (vs. 100% vPE) and yields a modest ~5% increase in impact strength (MFEsp). This outcome is consistent with Cestari et al. [37], who also observed improved impact strength in rotomolded composites upon adding up to 40–50% rPE.

At 50% rPE, however, internal stress rises sharply again—β increases by ~43% compared to its minimum at 30% rPE (about 7% higher than the 0% rPE case)—and accordingly the composite’s impact strength drops.

**Figure 20:**
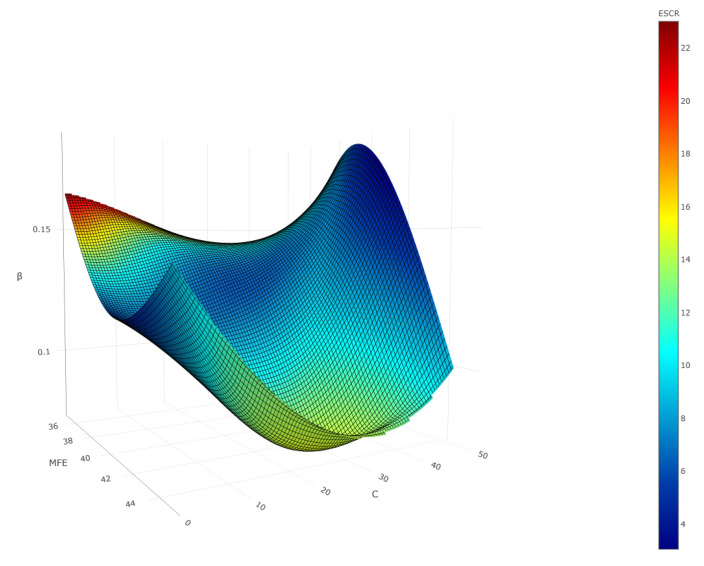
Correlation between MFEsp., ESCR, β, and the content of recycled polyethylene (rPE) in the studied rotational composites.

The correlation between MFEsp., ESCR, β, and rPE content for the studied rotational composites is presented in Figure 20.

Thus, using minimal internal stress as the criterion (β indicator), the optimal rPE content is about 30% or less (see Figure 18b).

Increasing pigment content up to ~0.2% raises internal stress (β) by ~27% before leveling off (Figure 18c). Concurrently, MFEsp drops by ~6% and then stabilizes. Based on the β criterion, ~0.2% pigment appears optimal for minimizing internal stress. However, since there is no significant difference in internal stress between 0.2% and 0.5% pigment, pigment concentration is not a critical factor in determining impact strength.

**Figure 21:**
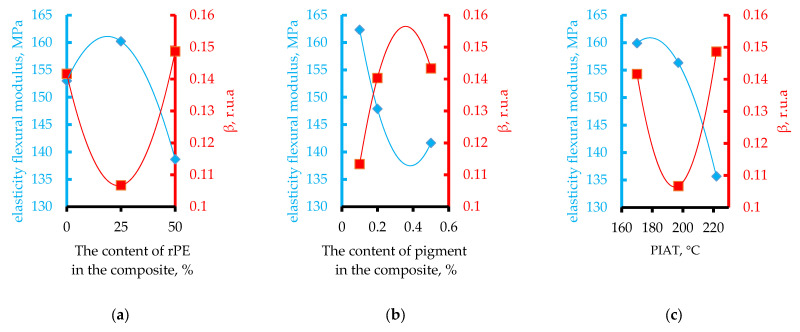
Correlation between the modulus of elasticity (E) and the height of the third harmonic (β) in rotational composites as a function of rPE content (**a**), pigment content (Cp) (**b**), and the PIAT parameter (**c**).

Of all the mechanical properties, only the elastic modulus E exhibited a clear inverse correlation with β (Figure 21).

This inverse relationship held true for variations in all three parameters (rPE, Cp, and PIAT)—whenever β changed due to these factors, the elastic modulus changed in the opposite direction. A possible explanation of this dependence may be the assumption that the modulus of elasticity characterizes the interaction between crystallites and amorphous regions in the composite. This assumption requires further study. Therefore, if maximizing the elastic modulus (and hence tank stiffness) is the goal, the optimal parameters would be roughly 20–30% rPE, 0.2% pigment, and a PIAT of 195 °C.

The other mechanical properties (yield strength, ultimate tensile strength, and elongation at yield) did not exhibit a strong correlation with β. Therefore, if one optimizes for maximum values of these properties (strength of the tank), the optimal rPE and pigment contents cannot be pinpointed unequivocally within the tested ranges (up to 50% rPE and 0.5% pigment. A possible explanation for the observed decrease in tensile strength and the simultaneous increase in elongation at break at elevated rPE contents (Figure 8a and Figure 9a) is a reduction in the overall crystallinity. Recycled polyethylene typically contains a higher fraction of amorphous regions and shorter, more heterogeneous chains, which disrupt the crystalline lamellae and weaken intermolecular packing. Reduced crystallinity lowers the tensile strength, whereas the more amorphous microstructure enhances ductility and, consequently, elongation at break. This hypothesis is consistent with the experimental trends observed in the present study.

PIAT proved to be a critical processing factor in rotational molding. Samples molded at 170 °C (insufficient sintering due to many micro-voids) or at 222 °C (thermal degradation) were deemed defective based on mechanical testing, ultrasonic inspection, and density measurements. Therefore, we recommend limiting the rotomolding process to a PIAT range of ~190–205 °C.

The IR spectroscopy results (Figure 16, showing no new oxygen-group peaks with more rPE) and contact angle measurements (Figure 12 and Figure 13) indicate that adding rPE has no statistically significant effect on the surface hydrophilicity of the PE. For instance, with 50% rPE, the contact angle θ dropped by only ~5%. Thus, incorporating up to 50% rPE does not meaningfully reduce the hydrophobicity of the PE tanks, and is unlikely to affect their service life. By contrast, increasing the pigment content (Figure 12b) does significantly reduce composite hydrophobicity, which can be attributed to the pigment’s high inherent hydrophilicity. This may adversely affect performance, as water molecules could be absorbed in the plastic’s micropores.

The influence of rPE on composite thermal stability is somewhat mixed. Adding up to 30% rPE causes slight increases in the thermal decomposition onset temperature Tb (~+1.5%) and peak decomposition temperature Tmax (~+0.1%), while slightly reducing the total mass loss M. In general, however, the addition of rPE has no major effect on the composites’ thermal stability. Adding pigment up to 0.5% produces small increases in Tb, Tmax, and M. Likewise, raising PIAT slightly improves the composite’s thermal stability. Therefore, if maximum thermal stability is the goal, the optimal parameters would be ~30% rPE, 0.5% pigment, and a PIAT of ~197 °C.

Summarizing the β, ESCR, mechanical, thermal, and impact performance results, we conclude that the optimal composite formulation for minimal internal stress and maximum overall performance is approximately 30% rPE, 0.5% pigment, and a PIAT of ~195 °C.

**Figure 22:**
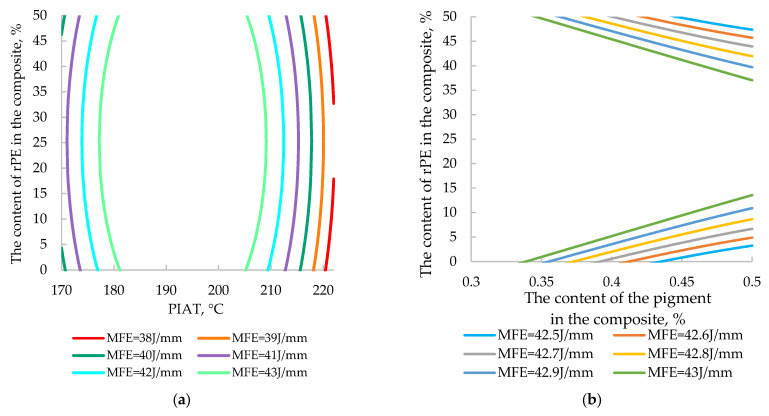
Two-factor nomograms: (**a**) rPE = *f* (MFE_sp._, PIAT), C_p._ = 0.5%; (**b**) rPE = *f* (MFE_sp._, C_p_), PIAT 195 °C.

**Figure 23:**
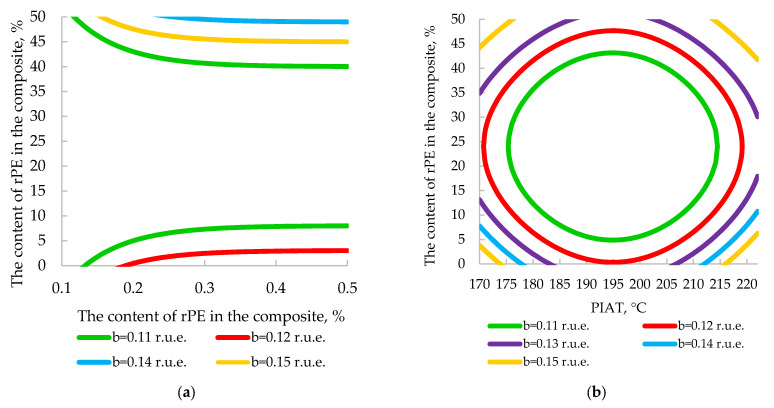
Two-factor nomograms: (**a**) rPE = *f* (*β*, PIAT), C_p._ = 0.5%; (**b**) rPE = *f* (*β*, C_p_), PIAT 195 °C.

At the request of AVAGRO’s technical department, we developed four nomograms (Figure 22 and Figure 23) to replace complex, labor-intensive calculations.

These nomograms enable quick estimation of a product’s impact strength and β based on the measured PIAT and the composite’s rPE and pigment contents, eliminating the need for tedious calculations. They also allow the inverse problem to be addressed—i.e., selecting the appropriate rPE and pigment levels for a desired impact strength or internal stress level (β). For example, when manufacturing a tank for storing liquid mineral fertilizer, MFEsp. must be at least 42 J/mm. To achieve this, it is necessary to strictly dose the rPE and pigment content (Figure 22b) in the composition and ensure the precise attainment of PIAT between 185 and 205 °C in the rotational molding cycle (Figure 22a).

This practical application of our model is especially noteworthy, as it offers a potential solution for a pressing issue: implementing rapid quality control of recycled rPE pellets in an industrial setting. Currently, the rotomolding industry lacks any quick method to assess the quality of incoming rPE batches. Manufacturers must mold trial cubes from the rPE, cut out 120 × 120 mm panels, condition them at –40 °C for 24 h, and then perform the MFEsp impact test. Such a procedure takes 2–7 days to complete. By contrast, when using ultrasonic testing, this time is significantly reduced because the molded part/cube does not need to be cut; it is sufficient to simply place the ultrasonic transducer against it. The value of β obtained within a few minutes is then compared with a reference value (similar to Figure 23). If β exceeds the allowable threshold, it indicates that internal stresses would drastically reduce the parts’ impact strength. This situation may result from severe thermal degradation of the rPE (due to multiple recycling cycles) or contamination with other plastics. In such cases, the proportion of recycled material should be reduced and β measured again until it falls within acceptable limits.

The observed correlation between the ultrasonic third-harmonic (β) and composite impact strength warrants further investigation via X-ray diffraction to clarify the underlying microstructural causes.

## 5. Conclusions

1. Our ESCR tests—a standard measure of internal stress in polyethylene—showed that the ultrasonic third-harmonic amplitude β is a viable quantitative indicator of internal stress in rotomolded parts. In fact, we found a strong correlation between β and all three key performance metrics.
2. IR spectroscopy and contact angle measurements showed that neither adding rPE (up to 50%) nor increasing PIAT caused any statistically significant increase in the hydrophilicity of PE. In contrast, raising the pigment concentration did measurably reduce the composites’ hydrophobicity.
3. Incorporating rPE had no appreciable effect on the composites’ thermal performance. Thus, using up to 50% recycled PE in these rotomolded composites is feasible for producing items with acceptable thermal stability. Adding pigment up to 0.5% gave a slight improvement in thermal stability, and a higher PIAT also modestly enhanced thermal resistance.
4. PIAT proved to be the dominant processing factor determining all the key performance characteristics of the rotomolded products. Therefore, we recommend limiting the PIAT to ~190–205 °C during processing of these composites.
5. Taking into account all mechanical and thermal results (including MFEsp, β, and ESCR), the composite formulation that minimizes internal stress—and thereby maximizes performance and service life—is ~30% rPE and 0.5% pigment, with a PIAT of ~195 °C.
6. In response to industry needs, we developed four nomograms (rPE = f(MFEsp, Cp, PIAT) and rPE = f(β, Cp, PIAT)) to eliminate tedious calculations. These nomograms enable rapid determination of a product’s impact strength and predicted β value based on the actual PIAT and the composite’s rPE and pigment contents. They can also be used inversely to choose appropriate rPE and pigment levels for a required impact strength or target internal stress (β).

## Figures and Tables

**Table 1:** Orthogonal experimental plan (3 factors × 3 levels) and results for each run: mechanical properties, thermal stability, ultrasonic third-harmonic amplitude β (r.u.a.), impact strength (MFEsp, J/mm), contact angle θ (degrees), and ESCR (hours).

n	Optimization Parameters			Obtained Experimental Parameters of Composites										
	rPE Content (%)	Pigment Content (%)	PIAT (°C)	Third-Harmonic Amplitude β (r.u.a.)	Mechanical Properties of the Obtained Composites				MFE_sp_ (J/mm)	Contact Angle θ (°)	ESCR (h)	Thermal Properties of the Obtained Composites		
					Max. Tensile Stress at Break (MPa)	Tensile Yield Stress (MPa)	Elongation at Break (%)	FLEXURAL Modulus E (MPa)				Onset Degradation Temp. Tb (°C)	Max Mass-Loss Temp. Tmax (°C)	Mass Loss M (%)
1	0	0.1	170	0.120	19.90	18.60	556.0	168	40.4	81.3	5	450	488.9	92.3
2	0	0.2	197	0.120	19.22	18.27	540.8	159	40	83.7	7	459	489.8	88.5
3	0	0.5	222	0.185	23.50	21.50	861.0	153	39	69.9	6.5	453	490.8	90.0
4	25	0.2	170	0.140	22.02	18.00	665.8	170	45	76.0	9	456	497.0	90.2
5	25	0.5	197	0.080	19.50	18.20	755.0	151	43	66.3	14	474	499.4	90.4
6	25	0.1	222	0.100	22.10	18.25	925.0	148	44.5	81.6	9	473	499.6	90.6
7	50	0.5	170	0.165	19.80	19.50	281.0	121	35	76.2	7	472	500.9	94.5
8	50	0.1	197	0.12	20.70	18.75	751.0	171	40	72.7	18	467	497.0	95.1
9	50	0.2	222	0.161	21.90	18.60	974.0	115	37	68.4	17	469	499.4	89.5

**Table 2:** Coefficient values used in Equations (3) and (4).

Estimated Parameter	*a*	*b*	*c*	*d*	*e*	*f*	*g*	*h*	*i*	*Y_M_*
MFE_sp_	−0.00136	0.0687	40.133	10.278	−12.75	42.806	−0.008367	3.2323	−267.89	40.43
*β*	0.00006	−0.0029	0.1417	0.145	−15.00	-	0.0000572	−0.0223	2.2789	0.13

## Data Availability

The original contributions presented in this study are included in the article/Supplementary Materials. Further inquiries can be directed to the corresponding author.

## Supplementary Materials

The following supporting information can be downloaded at: https://www.mdpi.com/article/10.3390/polym17243290/s1: Figure S1. Thermogravimetric (TG), derivative thermogravimetric (DTG) and differential thermal analysis (DTA) curves of Sample #1.; Figure S2. Thermogravimetric (TG), derivative thermogravimetric (DTG) and differential thermal analysis (DTA) curves of Sample #2.; Figure S3. Thermogravimetric (TG), derivative thermogravimetric (DTG) and differential thermal analysis (DTA) curves of Sample #3.; Figure S4. Thermogravimetric (TG), derivative thermogravimetric (DTG) and differential thermal analysis (DTA) curves of Sample #4.; Figure S5. Thermogravimetric (TG), derivative thermogravimetric (DTG) and differential thermal analysis (DTA) curves of Sample #5.; Figure S6. Thermogravimetric (TG), derivative thermogravimetric (DTG) and differential thermal analysis (DTA) curves of Sample #6.; Figure S7. Thermogravimetric (TG), derivative thermogravimetric (DTG) and differential thermal analysis (DTA) curves of Sample #7.; Figure S8. Thermogravimetric (TG), derivative thermogravimetric (DTG) and differential thermal analysis (DTA) curves of Sample #8.; Figure S9. Thermogravimetric (TG), derivative thermogravimetric (DTG) and differential thermal analysis (DTA) curves of Sample #9.; Figure S10. Comparative thermogravimetric curves of the composite samples, showing differences in mass loss and residual mass after heating.
