# Supplementary material for: Optimization of Composite Formulation Using Recycled Polyethylene for Rotational Molding
Source: Polymers (Basel). 2025 Dec 11;17(24):3290. doi: 10.3390/polym17243290 (PMC12736997; doi:10.3390/polym17243290)
Supplement: Supplementary file 1 [file polymers-17-03290-s001.zip › polymers-3931379-supplementary.pdf]

THERMAL ANALYSIS DATA
THERMOGRAVIMETRIC, DERIVATIVE THERMOGRAVIMETRIC AND DIFFERENTIAL THERMAL ANALYSIS CURVES

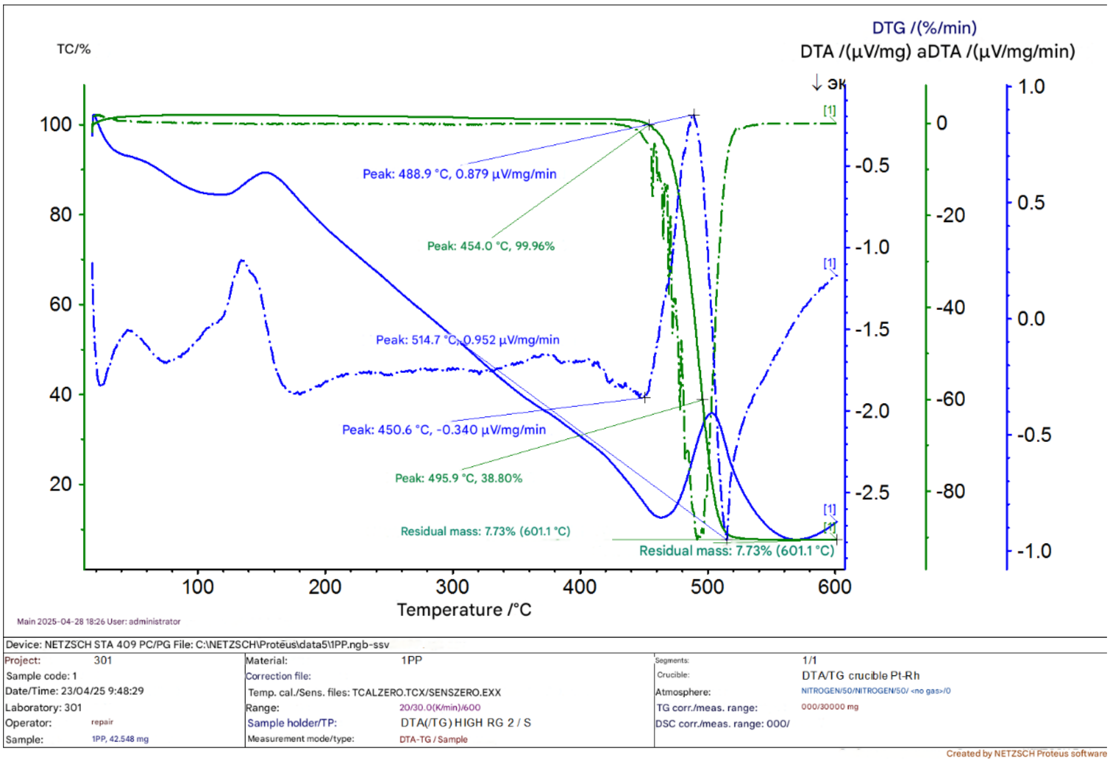

Figure S1. Thermogravimetric (TG), derivative thermogravimetric (DTG) and differential thermal analysis (DTA) curves of Sample #1.

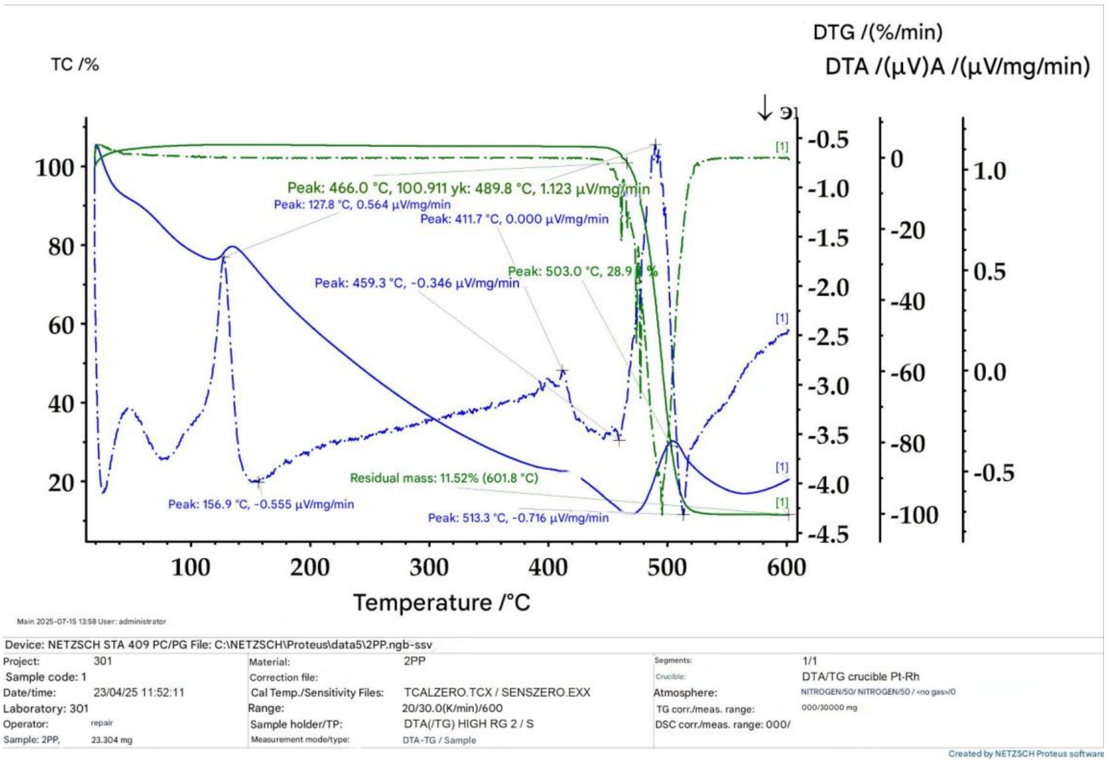

Figure S2. Thermogravimetric (TG), derivative thermogravimetric (DTG) and differential thermal analysis (DTA) curves of Sample #2.

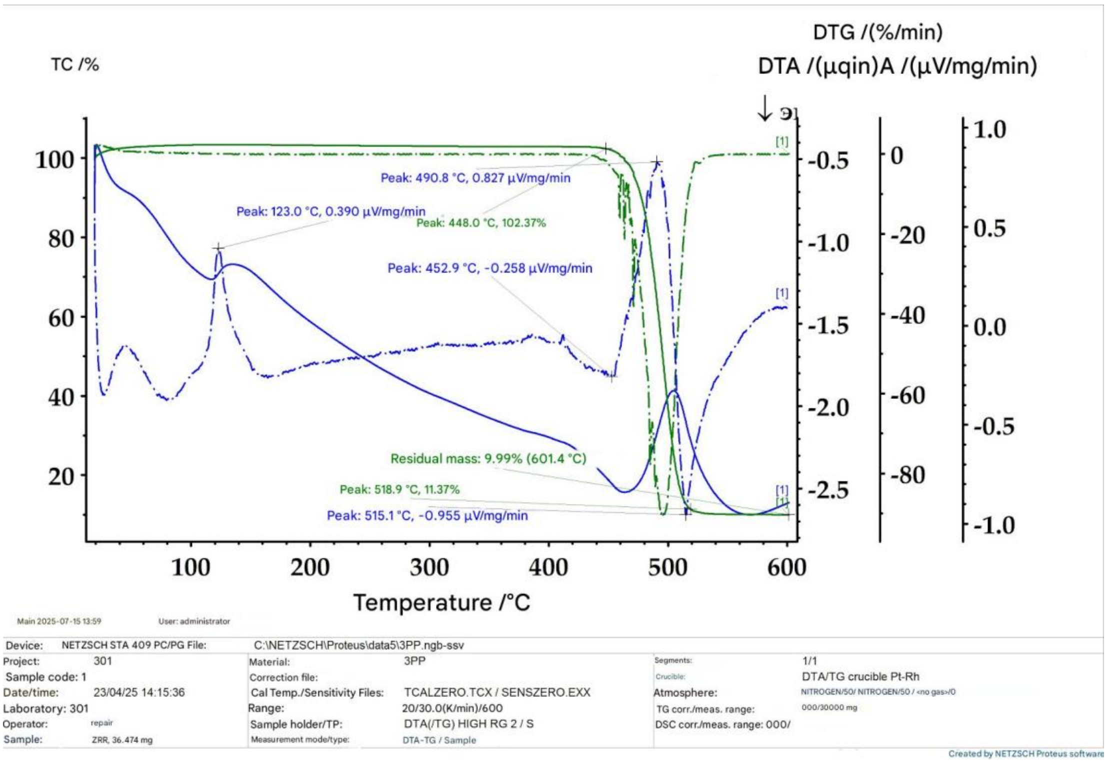

Figure S3. Thermogravimetric (TG), derivative thermogravimetric (DTG) and differential thermal analysis (DTA) curves of Sample #3.

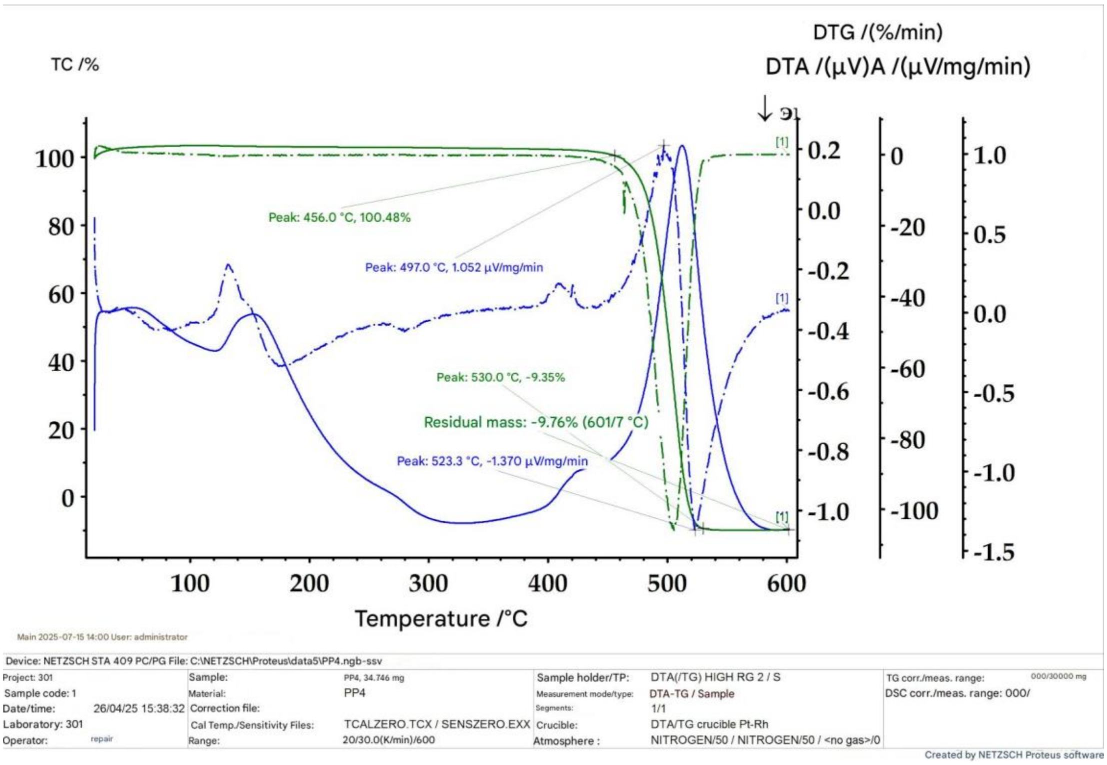

Figure S4. Thermogravimetric (TG), derivative thermogravimetric (DTG) and differential thermal analysis (DTA) curves of Sample #4.

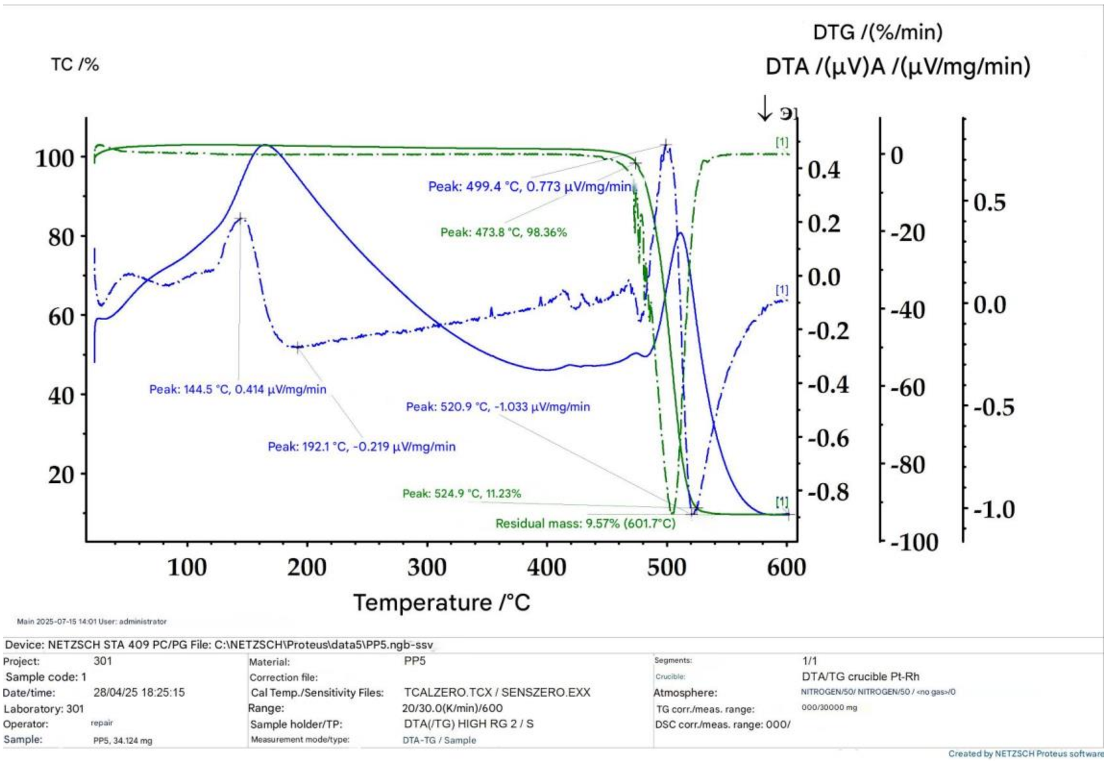

Figure S5. Thermogravimetric (TG), derivative thermogravimetric (DTG) and differential thermal analysis (DTA) curves of Sample #5.

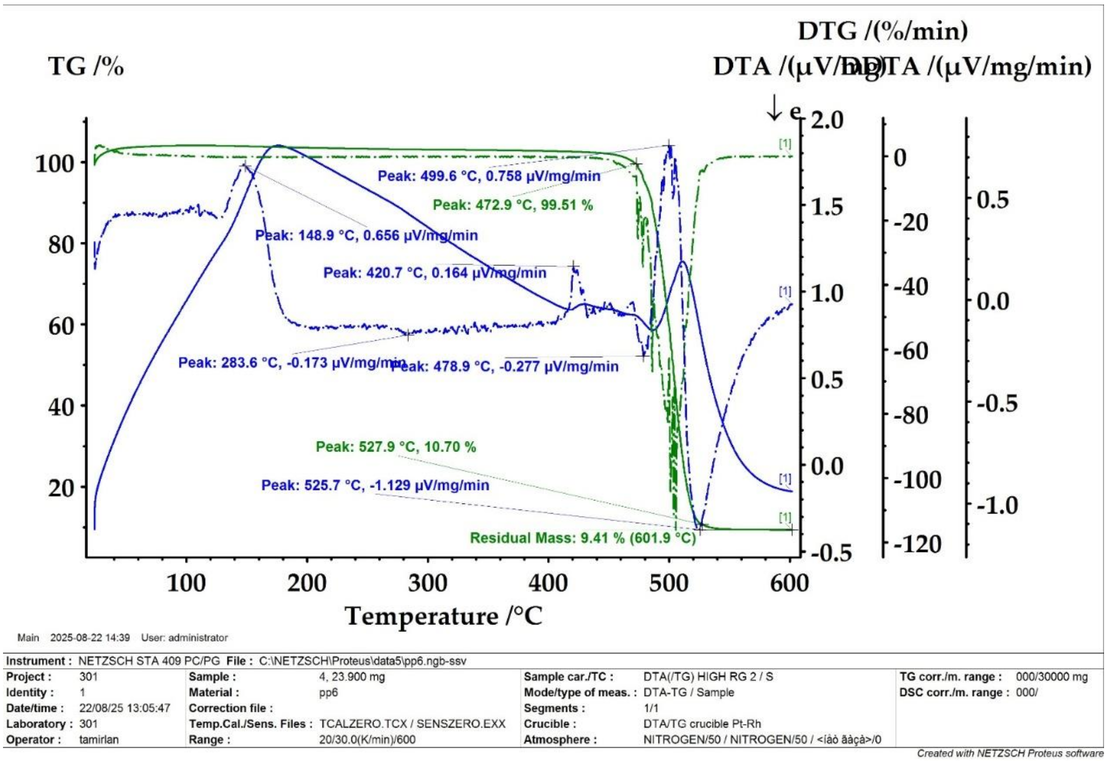

Figure S6. Thermogravimetric (TG), derivative thermogravimetric (DTG) and differential thermal analysis (DTA) curves of Sample #6.

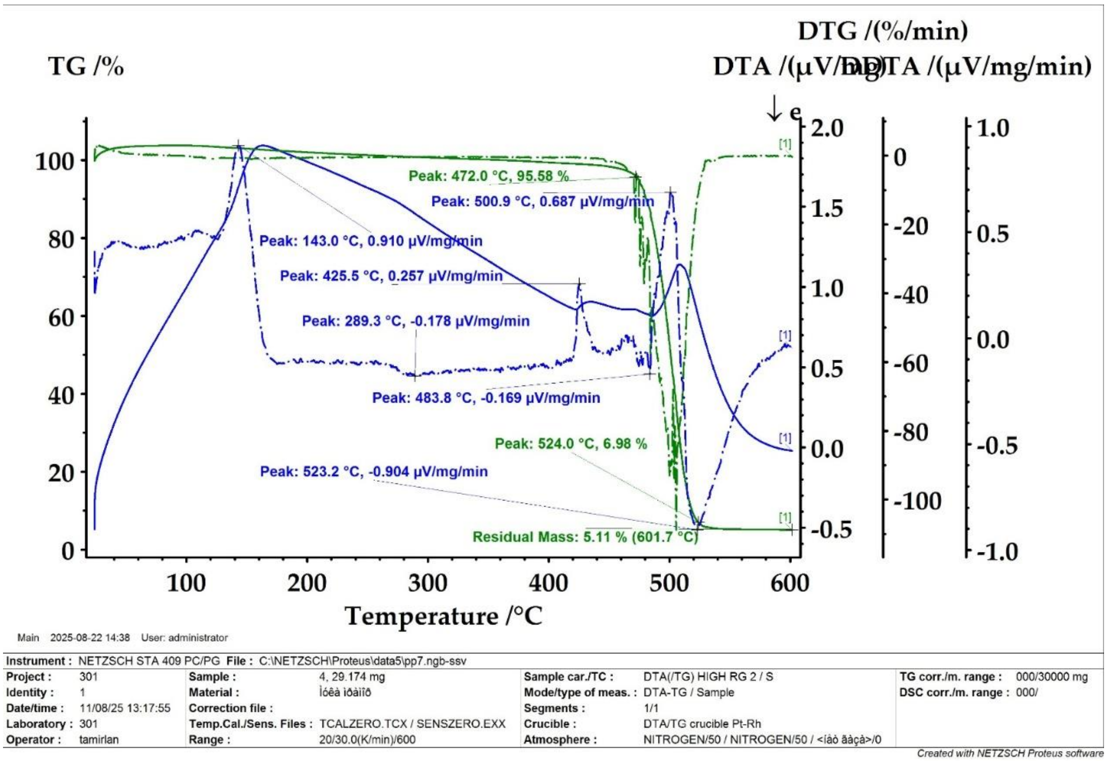

Figure S7. Thermogravimetric (TG), derivative thermogravimetric (DTG) and differential thermal analysis (DTA) curves of Sample #7.

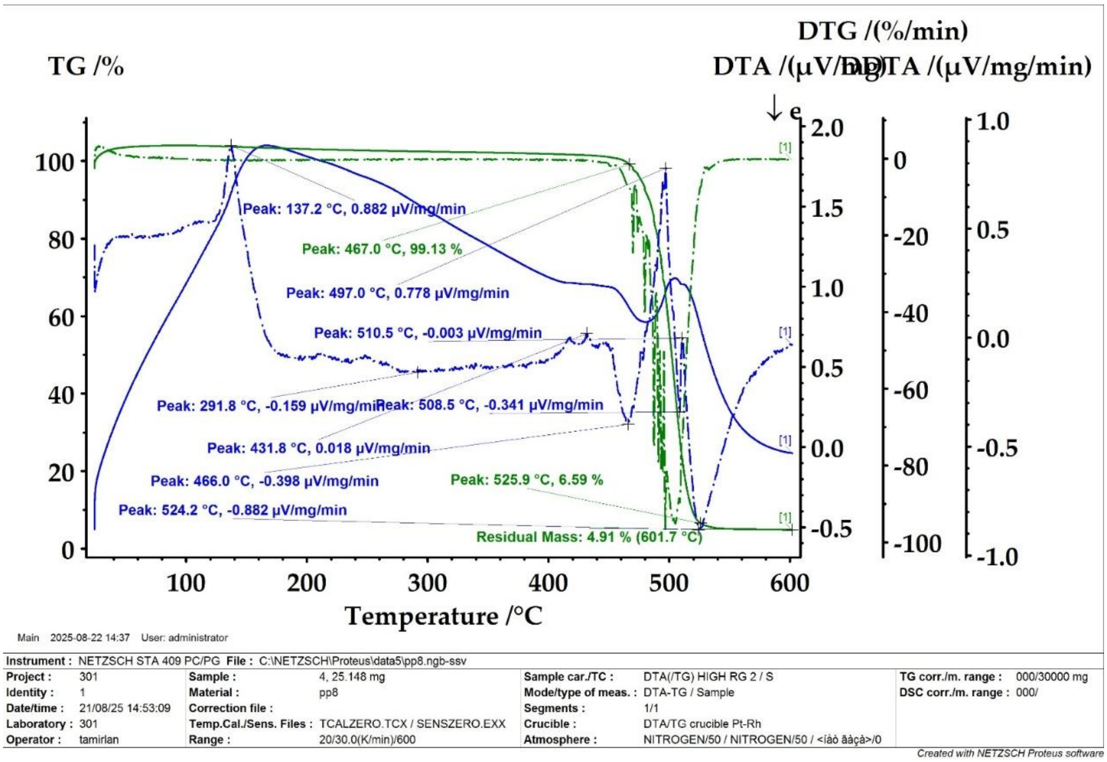

Figure S8. Thermogravimetric (TG), derivative thermogravimetric (DTG) and differential thermal analysis (DTA) curves of Sample #8.

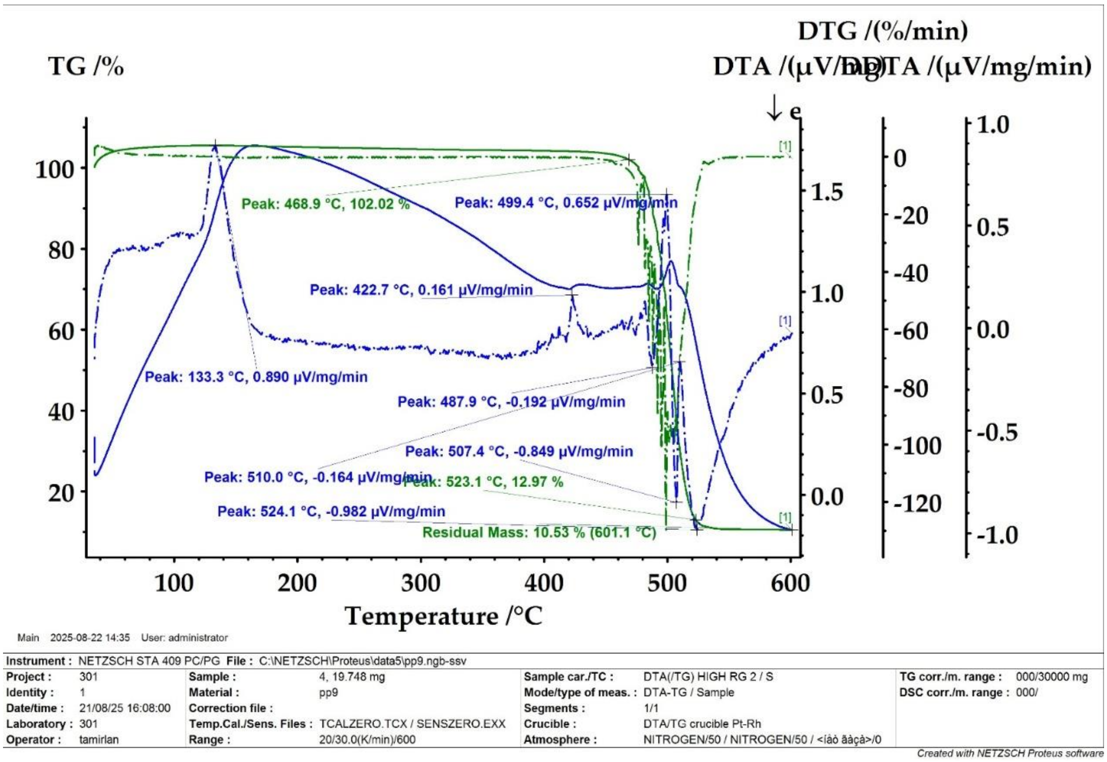

Figure S9. Thermogravimetric (TG), derivative thermogravimetric (DTG) and differential thermal analysis (DTA) curves of Sample #9.

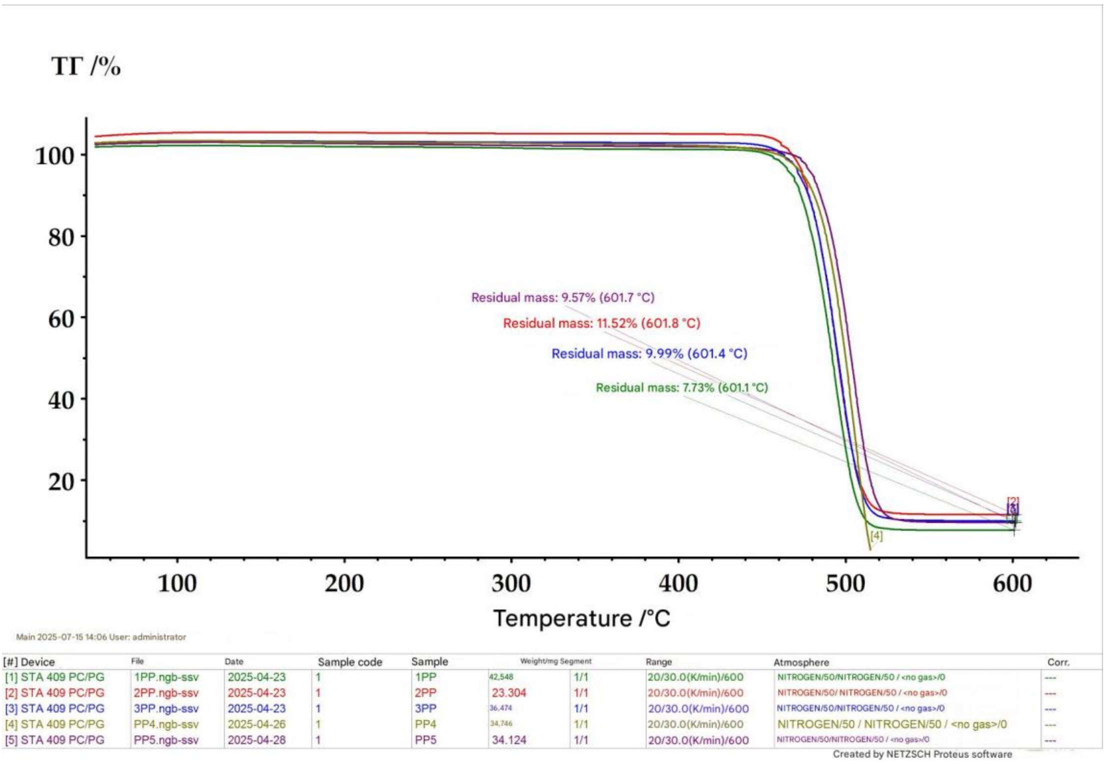

Figure S10. Comparative thermogravimetric curves of the composite samples, showing differences in mass loss and residual mass after heating
